# 25380 Cholecystokinin-B Receptor -Mediates Growth of Hepatocellular Carcinoma

**DOI:** 10.1017/cts.2021.427

**Published:** 2021-03-30

**Authors:** Martha Dee Gay, Anita Safronenka, Hong Cao, Robin Tucker, Narayan Shivapurkar, Jill P. Smith

**Affiliations:** Department of Medicine and Pathology, Georgetown University, Washington DC, USA

## Abstract

ABSTRACT IMPACT: Cholecystokinin-B Receptor -Mediates Growth of Hepatocellular Carcinoma with the use proglumide. Proglumide is a non-selective antagonistic drug therefore, strategies that block signaling at the CCK-BR may provide to be a novel therapeutic option for Hepatocellular Carcinoma treatment OBJECTIVES/GOALS: Cholecystokinin (CCK)and gastrin mediate the growth of Hepatocellular Carcinoma (HCC) through CCK-R and interruption of this signaling pathway could decrease HCC. CCK-Receptors are overexpressed in HCC and proliferation may be mediated through CCK-B. Blockade of the CCK-BR with proglumide decreased both growth in vitro and tumor growth in vivo. METHODS/STUDY POPULATION: RNA was extracted from murine Hepa1-6, RIL-175 and human HepG2 cells and was evaluated by qRT-PCR for expression of CCK-AR, CCK-BR and gastrin. CCK-R protein expression was analyzed by flow cytometry. HCC cells were treated in vitro with CCK peptide, the CCK-AR antagonist or the CCK-BR antagonist. Proliferation of selective CCK-R KO cells was compared to that of wild-type cells. To determine the effect of a CCK-R antagonist on tumor growth in vivo two cohorts of mice bearing subcutaneous Hepa1-6 or RIL-175 HCC tumors were treated with an oral bioavailable CCK-R antagonist proglumide or untreated water for 3-4 weeks. The mice bearing Hepa1-6 tumors were placed on a high-fat diet to raise blood CCK levels. Mice bearing RIL-175 tumors were fed standard chow to determine if proglumide could block autocrine growth by gastrin. RESULTS/ANTICIPATED RESULTS: The mRNA expression of CCK-AR, CCK-BR and gastrin were increased 80-90-fold in all HCC cell lines compared to that of normal liver. CCK-BRs were detected on >85% of the cells by flow cytometry. CCK peptide (1nM) stimulated HCC growth in vitro in both wild-type cells and in CCK-AR KO cells but not in CCK-BR KO cells. CCK-BR antagonist blocked CCK-stimulated growth in vitro but the CCK-AR antagonist did not, suggesting that the CCK-BR was responsible for mediating proliferation. In vivo tumor growth was significantly reduced with proglumide treatment by 70% (p<0.05) in Hepa1-6 and by 73% (p<0.001) in RIL-75 tumors, respectively. DISCUSSION/SIGNIFICANCE OF FINDINGS: CCK-Rs are overexpressed in HCC and proliferation appears to be mediated through the CCK-BR. Downregulation with CRISPR Cas9 or blockade of the CCK-BR with an antagonist decreases growth in vitro and proglumide therapy decreases tumor growth in vivo. Strategies that block signaling at the CCK-BR maybe a novel therapeutic option for HCC treatment.

